# Long-term outcomes and factors associated with revision after medial open-wedge high tibial osteotomy in 274 knees: a Kellgren–Lawrence grade-stratified analysis

**DOI:** 10.1186/s43019-026-00351-3

**Published:** 2026-10-20

**Authors:** Noriyuki Gomi, Nobuhiro Chikaishi, Ryuichi Isozaki

**Affiliations:** 1 Department of Joint Surgery Center, Sakaide Kaisei Hospital, Sakaide, Japan; 2 Department of Orthopaedic Surgery, Kagawa University, Miki, Japan

**Keywords:** Medial open-wedge high tibial osteotomy, Long-term outcomes, Kellgren–Lawrence grade, Revision, Revision risk, Joint line convergence angle

## Abstract

**Purpose:**

We aimed to investigate long-term outcomes and factors associated with revision after medial open-wedge high tibial osteotomy (OWHTO) according to Kellgren–Lawrence (KL) grade.

**Methods:**

This retrospective study included 274 knees (158 KL grade 4 [KL4] knees and 116 KL grades 2–3 [KL2–3] knees; mean age 64.7 ± 7.2 years) with a minimum follow-up of 10 years (mean follow-up, 12.5 ± 2.6 years) after OWHTO.

Revision surgery (conversion to total knee arthroplasty [TKA] or unicompartmental knee arthroplasty [UKA]) was defined as the endpoint. Clinical and radiographic outcomes were evaluated. Multivariate logistic regression analyses were performed separately for KL4 and KL2–3 knees to identify factors associated with revision.

**Results:**

The mean Japanese Orthopaedic Association (JOA) score improved from 60.6 preoperatively to 91.0 at plate removal, followed by gradual deterioration during long-term follow-up. Final JOA scores were significantly lower in KL4 knees than in KL2–3 knees (71.6 versus 83.1; *p* < 0.001). Revision surgery was performed in 43 knees (15.7%) and was significantly more frequent in KL4 than in KL2–3 knees (22.2% versus 6.9%; *p* < 0.01). Ten-year joint preservation rates were 90.5% in KL4 knees and 96.5% in KL2–3 knees (log-rank *p* = 0.006). In KL4 knees, postoperative percentage mechanical axis (%MA) at the time of plate removal was independently associated with revision risk (odds ratio [OR] 0.94, 95% confidence interval [CI] 0.91–0.98; *p* = 0.004). In KL2–3 knees, an exploratory multivariate analysis suggested an association between progression of joint line convergence angle during follow-up (ΔJLCA) and revision (OR 3.31, 95% CI 1.52–7.21; *p* = 0.003), whereas postoperative %MA showed no significant association.

**Conclusions:**

Long-term outcomes after OWHTO differed according to KL grade. KL4 knees demonstrated higher revision rates and inferior joint preservation survival, and postoperative weight-bearing line position was associated with revision risk. In contrast, revision was uncommon in KL2–3 knees, and exploratory analysis suggested a possible association with longitudinal changes in joint line convergence rather than postoperative alignment.

These findings suggest that factors associated with revision may differ according to disease severity.

**Level of evidence:**

This was a level IV, retrospective cohort study.

**Supplementary information:**

The online version contains supplementary material available at https://doi.org/10.1186/s43019-026-00351-3.

## Introduction

Open-wedge high tibial osteotomy (OWHTO) is an established joint-preserving procedure for medial compartment knee osteoarthritis (OA) and osteonecrosis (ON) [1], and its short-to-mid-term clinical outcomes have generally been reported as favorable [2, 3]. However, relatively few studies have reported outcomes beyond 10 years after OWHTO [4, 5], and the factors associated with long-term failure remain incompletely understood, particularly in advanced-stage disease. Previous studies have suggested that preoperative disease severity and postoperative alignment may influence long-term outcomes after OWHTO [3, 6]. Nevertheless, the relative importance of these factors in long-term failure remains controversial, particularly when stratified by disease severity. In addition, the time-dependent aspect of postoperative deterioration—namely, when and how clinical failure becomes apparent over long-term follow-up—has not been sufficiently investigated.

The Kellgren–Lawrence (KL) classification is widely used to assess radiographic osteoarthritis severity and has been widely applied in studies of knee osteoarthritis [7]. However, few studies have directly compared long-term clinical outcomes and factors associated with failure according to KL grade in OWHTO, and differences in factors associated with revision between advanced-stage and less advanced disease remain unclear [6].

Therefore, the purpose of this study was to investigate long-term outcomes and factors associated with revision after OWHTO according to Kellgren–Lawrence (KL) grade. Specifically, we sought to compare clinical outcomes, radiographic changes, survivorship, and factors associated with revision between KL grade 4 (KL4) and KL grades 2–3 (KL2–3) knees after more than 10 years of follow-up. We hypothesized that long-term outcomes and factors associated with revision would differ according to osteoarthritis severity. Clarifying these differences may contribute to a better understanding of factors associated with long-term revision after OWHTO.

## Methods

### Study design and patients

This retrospective cohort study included patients who underwent medial open-wedge high tibial osteotomy (OWHTO) for medial compartment knee osteoarthritis (OA) or osteonecrosis (ON) at our institution between June 2007 and May 2016.

The inclusion criteria were: (1) medial compartment OA or ON treated with OWHTO, (2) availability of complete clinical and radiographic data, and (3) a minimum postoperative follow-up of 10 years or revision surgery during the follow-up period.

The exclusion criteria were: (1) inflammatory arthritis, (2) previous knee arthroplasty, (3) advanced osteoarthritis in the lateral compartment, (4) severe patellofemoral osteoarthritis requiring alternative procedures, and (5) insufficient clinical or radiographic follow-up.

**Fig. 1:**
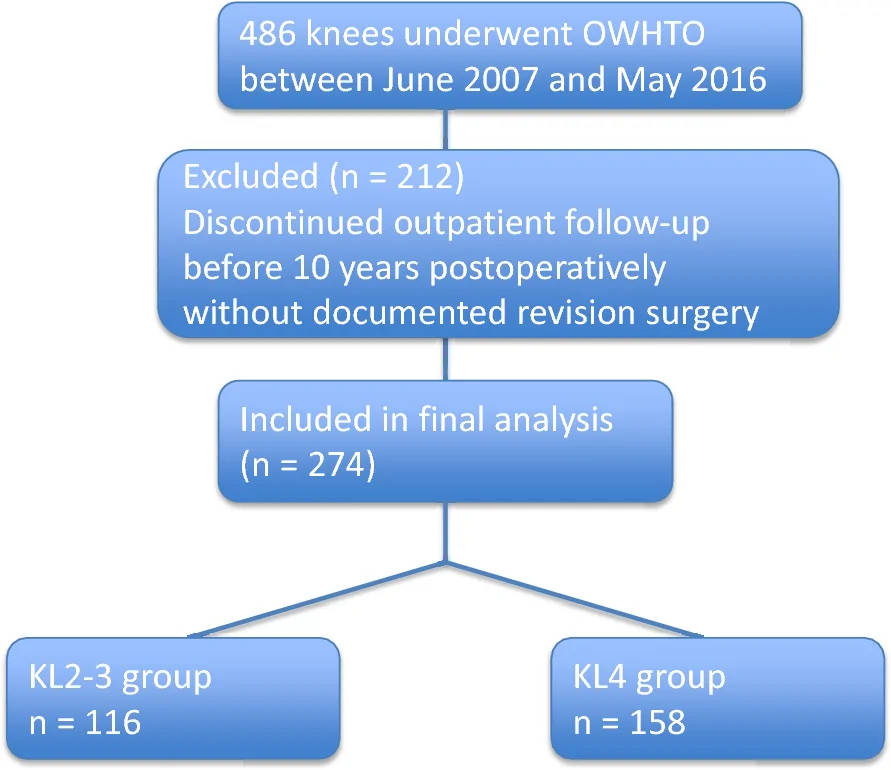
Patient flow diagram of study enrollment

Of the 486 knees that underwent OWHTO during the study period, 274 met the inclusion criteria and were included in the final analysis. These included all knees with a minimum follow-up of 10 years as well as knees that underwent revision surgery during the follow-up period. The remaining 212 knees were excluded because outpatient follow-up was discontinued before 10 years postoperatively, and no revision surgery had been documented. The patient selection process is summarized in Fig. 1.

Radiographic severity was assessed using the Kellgren–Lawrence (KL) grading system. Patients were stratified into two groups: KL grade 4 (KL4) and KL grades 2–3 (KL2–3). Concomitant ligament instability requiring surgical treatment was uncommon in this cohort, with only one patient undergoing combined anterior cruciate ligament (ACL) reconstruction.

### Surgical technique and postoperative management

OWHTO was performed using a standard medial approach.

After a curved skin incision on the medial aspect of the proximal tibia, the superficial medial collateral ligament was partially released. A biplanar osteotomy was created from the medial proximal tibia toward the center of the proximal tibiofibular joint under fluoroscopic guidance, leaving the lateral cortex intact as a hinge.

The osteotomy gap was gradually opened to achieve the planned correction, and fixation was performed using a locking plate system (TomoFix^®^, DePuy Synthes).

The osteotomy gap was filled with either autologous iliac crest bone graft or β-tricalcium phosphate bone substitute, according to surgeon preference. The surgical procedure was performed in accordance with the techniques described by Lobenhoffer and Staubli [1, 8].

Postoperatively, range-of-motion exercises were initiated within the first postoperative week. Partial weight-bearing was allowed at 2 weeks after surgery, followed by progression to full weight-bearing at approximately 4–6 weeks, depending on radiographic evidence of bone healing.

### Preoperative planning and alignment target

Preoperative planning was performed using standing full-length anteroposterior radiographs of both lower extremities. The correction target was defined as a mechanical axis passing through 70% of the lateral tibial plateau, measured from the medial edge.

The correction angle and opening width were calculated according to the Ogata–Miniaci method [9, 10]. Although a uniform target of approximately 70% was used, actual postoperative alignment varied among patients, enabling subgroup analysis based on achieved alignment. Alignment targets were not individualized according to preoperative KL grade. Therefore, the present study evaluated long-term outcomes and factors associated with revision in a cohort treated with a uniform correction target and was not designed to compare predefined alignment strategies.

### Clinical and radiographic evaluation

Clinical outcomes were assessed using the Japanese Orthopaedic Association (JOA) knee score [11], a validated clinical scoring system widely used in Japan to evaluate knee function in patients with osteoarthritis. The score evaluates four domains (pain on walking, pain on ascending and descending stairs, range of motion, and joint effusion), with a maximum score of 100 points, indicating better knee function. Knee flexion and extension range of motion were also recorded. Evaluations were performed preoperatively, at the time of plate removal (mean 15.3 months postoperatively; range, 7.7–37.5 months), at 5 years, and at final follow-up. Plate removal was generally performed after radiographic confirmation of bone union. The institutional criteria for plate removal were bridging bone formation over more than half of the osteotomy gap on the anteroposterior radiograph together with union of the anterior and posterior cortices on the lateral radiograph. In exceptional cases, plate removal was performed earlier because of implant-related infection or later because of patient preferences or scheduling considerations.

Radiographic evaluation included measurement of the percentage mechanical axis (%MA), joint line convergence angle (JLCA), medial proximal tibial angle (MPTA), femorotibial angle (FTA), and mechanical lateral distal femoral angle (mLDFA). All radiographic measurements were performed by a single experienced orthopedic surgeon using standardized measurement methods on standing radiographs. Radiographic images were imported into dedicated planning software (OrthoPlanner Pro; TOYO Corporation, Tokyo, Japan), calibrated before measurement, and analyzed using predefined anatomical landmarks and measurement protocols provided by the software. To minimize measurement variability, all measurements were performed using the same protocol throughout the study. Radiographic evaluations were performed at the same time points as the clinical assessments. To assess intraobserver reliability, 30 knees (15 KL4 and 15 KL2–3 knees) were randomly selected and remeasured by the same observer using the same measurement protocol. Intraclass correlation coefficients (ICCs) were calculated for postoperative %MA, JLCA at plate removal, JLCA at final follow-up, and ΔJLCA. KL grade was determined based on Rosenberg views and standing full-length radiographs, and the more advanced medial joint space narrowing was adopted when discrepancies were present.

### Definition of alignment changes

Changes in alignment parameters were calculated using the values at the time of plate removal as the reference. For %MA and MPTA, delta values were calculated as the plate removal value minus the final follow-up value, such that positive values represented loss of correction over time. For JLCA and FTA, delta values were calculated as the final follow-up value minus the plate removal value. The time of plate removal was selected as the reference point because bone healing and correction alignment were considered to be stabilized at this stage, allowing subsequent longitudinal changes during long-term follow-up to be evaluated.

### Definition of revision and survivorship analysis

Revision was defined as conversion to total knee arthroplasty (TKA) or unicompartmental knee arthroplasty (UKA) for any reason.

Survivorship was analyzed using the Kaplan–Meier method, with revision surgery as the endpoint. Patients who did not undergo revision were censored at the time of the final follow-up. Survivorship curves were compared between groups using the log-rank test.

### Exploratory subgroup analysis according to achieved postoperative alignment

Because actual postoperative alignment varied despite the use of a uniform correction target, an exploratory subgroup analysis was performed according to the achieved postoperative %MA at the time of plate removal:

- < 60%
- 60–65%
- > 65%

Revision rates, final clinical outcomes (JOA score and range of motion), and postoperative alignment changes from plate removal to final follow-up (Δ%MA and ΔJLCA) were compared among the three groups.

This exploratory subgroup analysis was performed primarily in the KL4 group and secondarily in the KL2–3 group using the same methodology.

### Statistical analysis

Continuous variables are presented as mean ± standard deviation and categorical variables as number and percentage. Group comparisons were performed using the Mann–Whitney *U* test or Kruskal–Wallis test for continuous variables and the chi-squared test for categorical variables. When significant differences were detected among three groups, post hoc multiple comparisons were performed using the Dunn method with Bonferroni correction. Multivariate logistic regression analysis was conducted to identify factors independently associated with revision. Variables entered into the model included postoperative %MA at the time of plate removal, ΔJLCA, age, and body mass index (BMI), selected based on prior literature and clinical relevance while avoiding collinearity with other alignment parameters. Odds ratios (ORs) with 95% confidence intervals (CIs) were calculated.

For subgroup analysis in the KL4 group, patients were stratified according to postoperative %MA at the time of plate removal into three categories (< 60%, 60–65%, and > 65%) based on postoperative alignment ranges commonly reported in previous OWHTO studies.

All statistical analyses were performed using IBM SPSS Statistics (version 30.0; IBM Corp., Armonk, NY, USA), and *p* < 0.05 was considered statistically significant. Cases with missing data for any variable included in the multivariate model were excluded from the respective analyses.

Because this was a retrospective study including all eligible cases with a minimum follow-up of 10 years, no a priori sample size calculation was performed.

## Results

### Patient characteristics

Of the 486 knees that underwent OWHTO during the study period, 274 knees met the inclusion criteria and were included in the final analysis. The mean age at the time of surgery was 64.7 ± 7.2 years. Of the 274 knees, 207 (75.5%) were from female patients.

The mean body mass index (BMI) was 26.5 ± 3.8 kg/m^2^. There were 158 and 116 knees in the KL4 and KL2–3 groups, respectively. Baseline demographic characteristics are summarized in Table 1. Age, sex distribution, BMI, and follow-up duration did not differ significantly between the two groups. As expected, the distribution of OA and ON differed significantly because patients were stratified according to radiographic disease severity.

**Table 1:** Baseline demographic characteristics

Variable	KL2–3 (*n* = 116)	KL4 (*n* = 158)	*p*-Value
Age (years)	64.30 ± 7.80	64.97 ± 6.80	0.459
Sex (female/male)	82/34	125/33	0.144
BMI (kg/m^2^)	26.14 ± 3.39	26.82 ± 4.03	0.131
Follow-up (years)	12.34 ± 2.55	12.08 ± 2.58	0.407
Diagnosis (OA/ON)	72/44	154/4	< 0.001

*BMI* body mass index, *OA* osteoarthritis, *ON* osteonecrosis

The mean observation period for the entire cohort was 12.5 ± 2.6 years (range, 2.1–18.3 years).

Baseline characteristics of patients lost to follow-up are summarized in Supplementary Table S2. Patients lost to follow-up were significantly older and more frequently male than those included in the final analysis. However, no significant differences were observed in BMI, diagnosis (OA/ON), or KL grade distribution between the two groups.

### Clinical outcomes

The mean JOA score for all knees improved from 60.6 points preoperatively to 91.0 points at the time of plate removal. Thereafter, the score declined to 86.7 points at 5 years and further decreased to 76.5 points at the final follow-up.

In the KL2–3 group, the mean JOA score was 94.2 points at the time of plate removal and 83.1 points at the final follow-up, whereas in the KL4 group, the corresponding values were 88.6 points at the time of plate removal and 71.6 points at the final follow-up. The JOA score at the final follow-up was significantly lower in the KL4 group than in the KL2–3 group (*p* < 0.001).

**Fig. 2:**
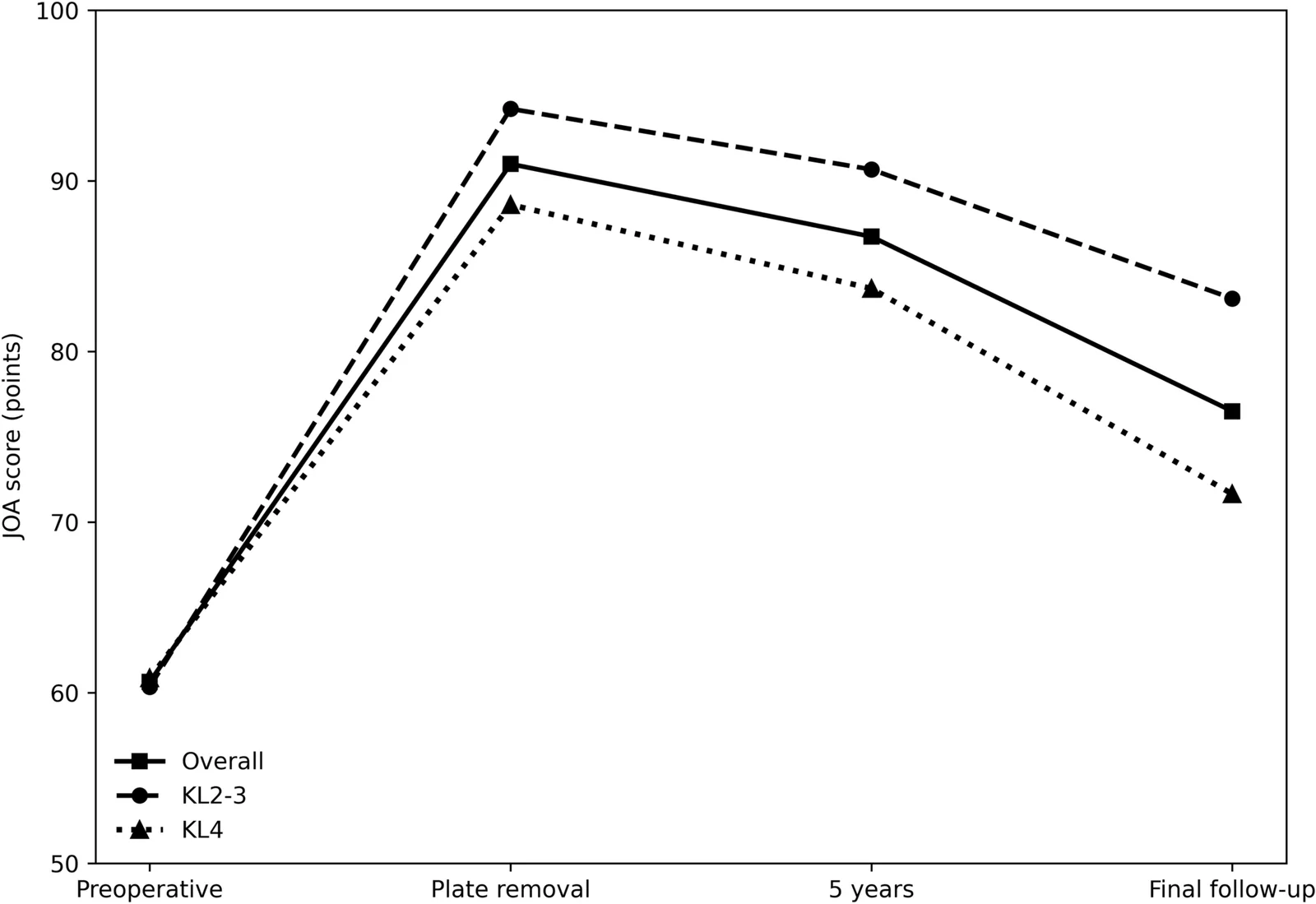
Time course of JOA score changes according to KL grade. JOA scores improved markedly in both groups at the time of plate removal after OWHTO. A gradual decline was observed between plate removal and 5 years postoperatively, with no pronounced difference between groups at 5 years. During long-term follow-up beyond 5 years, clinical deterioration became more evident, particularly in the KL4 group, resulting in significantly lower JOA scores at final follow-up (*p* < 0.001)

Although both groups showed substantial postoperative improvement, the decline in JOA score during long-term follow-up was more pronounced in the KL4 group. The time course of JOA score changes according to KL grade is illustrated in Fig. 2, demonstrating an earlier and more pronounced decline in the KL4 group.

At the time of plate removal, mean knee flexion was 131.8° in the KL2–3 group and 125.1° in the KL4 group, and at the final follow-up, it was 130.0° and 114.2°, respectively. Final knee flexion was significantly lower in the KL4 group (*p* < 0.001).

Mean knee extension at plate removal was −1.1° in the KL2–3 group and −3.2° in the KL4 group, and at the final follow-up, it was −1.3° and −4.6°, respectively. At the final follow-up, extension limitation was significantly more pronounced in the KL4 group (*p* = 0.018).

### Radiographic outcomes

#### Radiographic alignment assessment

Radiographic alignment parameters measured preoperatively, at the time of plate removal, and at the final follow-up are summarized in Table 2.

**Table 2:** Radiographic alignment parameters at each point

Parameter	KL2–3 pre	KL2–3 plate removal	KL2–3 final	KL4 pre	KL4 plate removal	KL4 final
%MA (%)	26.4	67.5	63.4	13.2	70.1	60.7
JLCA (°)	3.41	2.60	2.94	6.16	3.86	4.94
MPTA (°)	85.98	95.05	94.16	85.79	97.27	95.98
FTA (°)	179.33	169.11	170.23	182.57	168.52	171.02
mLDFA (°)	87.9	—	—	88.3	—	—

Values are presented as mean values

Angular measurements are expressed in degrees

mLDFA was measured only preoperatively

*%MA* percentage mechanical axis, *JLCA* joint line convergence angle, *MPTA* medial proximal tibial angle, *FTA* femorotibial angle, *mLDFA* mechanical lateral distal femoral angle

%MA shifted laterally after surgery in both groups, reaching approximately 70% at the time of plate removal. A subsequent medial shift was observed at the final follow-up, with a significantly greater correction loss in the KL4 group than in the KL2–3 group (Δ%MA: 9.09 ± 14.65% versus 4.10 ± 11.02%; *p* = 0.001). JLCA decreased after surgery in both groups but increased again at the final follow-up, and the increase was significantly greater in the KL4 group (ΔJLCA: 1.15 ± 2.22° versus 0.33 ± 1.92°; *p* = 0.003). MPTA increased after surgery in both groups and remained relatively stable during follow-up.

FTA decreased after surgery in both groups and increased slightly at the final follow-up, particularly in the KL4 group.

Changes in alignment parameters from the time of plate removal to the final follow-up are presented in Table 3. Correction loss, represented by Δ%MA, was significantly greater in the KL4 group (9.09%) than in the KL2–3 group (4.10%) (*p* = 0.001). Similarly, the increase in JLCA during follow-up (ΔJLCA) was significantly greater in the KL4 group (1.15°) than in the KL2–3 group (0.33°) (*p* = 0.003). ΔFTA was also significantly greater in the KL4 group (2.43°) than in the KL2–3 group (1.19°) (*p* = 0.001). In contrast, no significant difference was observed between groups for ΔMPTA (*p* = 0.251).

**Table 3:** Changes in alignment parameters from the time of plate removal to final follow-up

Parameter	KL2–3 (Δ)	KL4 (Δ)	*p*-Value
Δ%MA (%)	4.10 ± 11.02	9.09 ± 14.65	0.001
ΔJLCA (°)	0.33 ± 1.92	1.15 ± 2.22	0.003
ΔMPTA (°)	0.94 ± 2.05	1.19 ± 2.44	0.251
ΔFTA (°)	1.19 ± 2.97	2.43 ± 3.65	0.001

Values are presented as mean ± standard deviation. For %MA and MPTA, positive Δ values indicate loss of correction from plate removal to final follow-up. For JLCA and FTA, Δ values were calculated as the final follow-up value minus the plate removal value

*%MA* percentage mechanical axis, *JLCA* joint line convergence angle, *MPTA* medial proximal tibial angle, *FTA* femorotibial angle

To assess measurement reliability, intraobserver reproducibility was evaluated in 30 randomly selected knees. The ICCs were 0.970 for postoperative %MA, 0.929 for JLCA at plate removal, 0.885 for JLCA at final follow-up, and 0.764 for ΔJLCA, indicating good-to-excellent reliability.

### Revision and survivorship

Overall, 43 knees (15.7%) underwent revision surgery, including 32 TKA and 11 UKA. The mean time from OWHTO to revision surgery was 9.9 ± 3.1 years.

The revision rate was 6.9% (8 of 116 knees) in the KL2–3 group, compared with 22.2% (35 of 158 knees) in the KL4 group, indicating a significantly higher revision rate in the KL4 group (*p* < 0.01).

The most common cause of revision was recurrence of medial compartment osteoarthritis (34 knees), followed by progression of lateral compartment osteoarthritis (3 knees), global progression of osteoarthritis (3 knees), recurrent varus deformity due to plate failure (2 knees), and recurrence of osteonecrosis (1 knee). No revisions were attributed to patellofemoral joint deterioration.

**Fig. 3:**
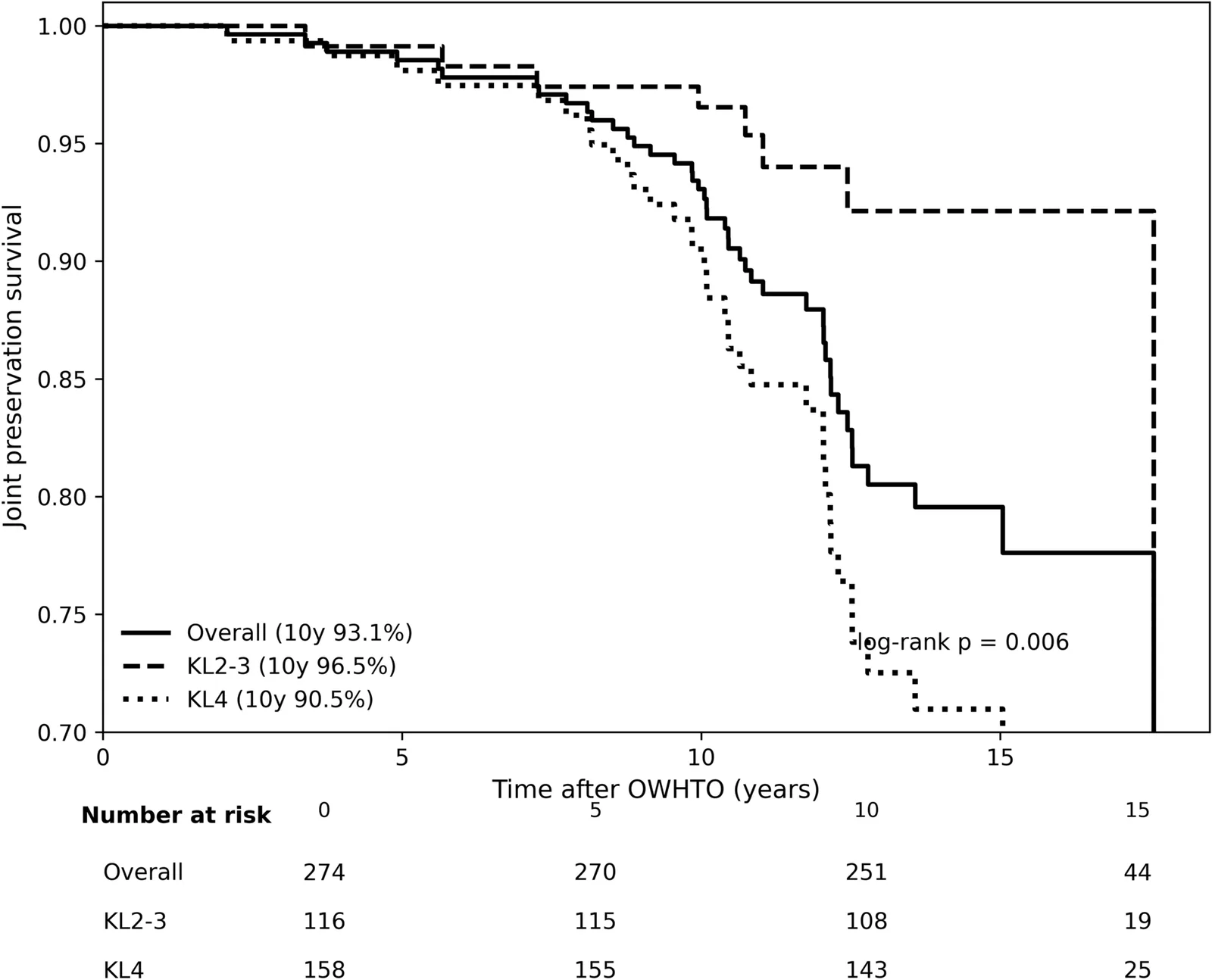
Kaplan–Meier survival curves for joint preservation according to KL grade. The KL4 group demonstrated significantly lower joint preservation survival than the KL2–3 group (log-rank *p* = 0.006). Numbers at risk are shown below the graph

Kaplan–Meier survival analysis demonstrated that joint preservation rates at 10 years were 96.5% in the KL2–3 group and 90.5% in the KL4 group. Joint preservation survival was significantly lower in the KL4 group than in the KL2–3 group (log-rank *p* = 0.006) (Fig. 3).

### Multivariate analysis (KL-specific analysis)

Multivariate logistic regression analysis was performed with revision surgery as the dependent variable and postoperative %MA at the time of plate removal, ΔJLCA, age, and BMI as independent variables.

In the KL4 group, postoperative %MA at the time of plate removal was independently associated with revision (odds ratio [OR] 0.94; 95% confidence interval [CI] 0.91–0.98; *p* = 0.004). A more laterally positioned postoperative weight-bearing line was associated with a lower likelihood of revision. In contrast, ΔJLCA, age, and BMI were not significantly associated with revision in the multivariate model (Table 4).

**Table 4:** Multivariate logistic regression analysis for revision in the KL4 group

Variable	Odds ratio	95% confidence interval	*p*-Value
%MA at plate removal	0.94	0.91–0.98	0.004
ΔJLCA (°)	1.21	0.97–1.52	0.089
Age (years)	1.02	0.97–1.07	0.41
BMI (kg/m^2^)	1.05	0.96–1.16	0.29

Values were obtained using multivariate logistic regression analysis

Revision was defined as conversion to UKA or TKA

Dependent variable: revision (conversion to UKA or TKA)

Independent variables: postoperative %MA at the time of plate removal, ΔJLCA, age, BMI

*%MA* percentage mechanical axis, *JLCA* joint line convergence angle, *BMI* body mass index, *UKA* unicompartmental knee arthroplasty, *TKA* total knee arthroplasty

### Subgroup analysis according to postoperative %MA at the time of plate removal in the KL4 group

When patients in the KL4 group were stratified according to postoperative %MA at the time of plate removal (< 60%, 60–65%, and > 65%), revision rates were 41.4% (12/29 knees), 23.1% (6/26 knees), and 16.5% (17/103 knees), respectively (Table 5). Revision rates decreased progressively with increasing postoperative %MA.

**Table 5:** Final clinical outcomes and alignment changes according to postoperative %MA at the time of plate removal in the KL4 group

Variable	< 60% (*n* = 29)	60–65% (*n* = 26)	> 65% (*n* = 103)	*p*-Value
Revision, *n* (%)	12/29 (41.4%)	6/26 (23.1%)	17/103 (16.5%)	0.017
Final JOA score (points)	66.6 ± 11.5	72.5 ± 12.4	72.9 ± 12.3	0.041
Final flexion (°)	111.0 ± 11.8	116.5 ± 8.6	115.0 ± 11.3	0.164
Final extension (°)	−3.8 ± 3.4	−2.3 ± 2.9	−3.0 ± 3.3	0.244
Δ%MA (%)	14.2 ± 11.5	10.6 ± 11.0	7.2 ± 16.0	0.019
ΔJLCA (°)	1.2 ± 2.1	1.0 ± 2.4	1.2 ± 2.2	0.999

Values are presented as mean ± standard deviation or *n* (%). Revision was defined as conversion to UKA or TKA

For %MA, positive Δ values indicate loss of correction from plate removal to final follow-up. ΔJLCA was calculated as the final follow-up value minus the plate removal value

*p*-Values represent comparisons among the three groups (continuous variables: Kruskal–Wallis test; categorical variables: chi-squared test)

*JOA* Japanese Orthopaedic Association, *%MA* percentage mechanical axis, *JLCA* joint line convergence angle, *UKA* unicompartmental knee arthroplasty, *TKA* total knee arthroplasty

Final JOA scores differed significantly among the three groups (*p* = 0.041), with the < 60% group demonstrating lower scores than the other two groups. No significant difference was observed between the 60–65% and > 65% groups.

Regarding alignment changes from the time of plate removal to final follow-up, Δ%MA differed significantly among the three groups (*p* = 0.019), with lower Δ%MA values observed in the groups with greater postoperative lateralization. No significant difference in ΔJLCA was observed among the three groups (Table 5).

### Multivariate analysis in the KL2–3 group

In the KL2–3 group, an exploratory multivariate logistic regression analysis (Supplementary Table S1) was performed despite the limited number of revision events and suggested that ΔJLCA was associated with revision (OR 3.31; 95% CI 1.52–7.21; *p* = 0.003). In contrast, postoperative %MA at the time of plate removal, age, and BMI were not significantly associated with revision.

As an exploratory analysis to further investigate factors associated with ΔJLCA progression, the relationship between ΔJLCA and preoperative alignment parameters was evaluated. Preoperative MPTA showed a weak positive correlation with ΔJLCA (*r* = 0.22), whereas mLDFA demonstrated a weaker correlation (*r* = 0.10).

In the KL2–3 group, patients were stratified according to postoperative %MA at the time of plate removal into three groups: < 60%, 60–65%, and > 65%, comprising 27, 18, and 71 knees, respectively.

Revision surgery (conversion to UKA or TKA) was performed in 4 of 27 knees (14.8%) in the < 60% group, 0 of 18 knees (0%) in the 60–65% group, and 4 of 71 knees (5.6%) in the > 65% group, with no significant difference among the groups (chi-squared test; *p* = 0.126).

There were also no significant intergroup differences in final JOA score, final knee flexion, final knee extension, or postoperative alignment changes from the time of plate removal to final follow-up (Δ%MA and ΔJLCA) (Table 6).

**Table 6:** Comparison of outcomes according to postoperative %MA at the time of plate removal in the KL2–3 group

Variable	< 60%	60–65%	> 65%	*p*-Value
Number of knees, *n*	27	18	71	—
Revision, *n* (%)	4 (14.8%)	0 (0.0%)	4 (5.6%)	0.126
Final JOA score (points)	82.2 ± 12.7	87.5 ± 13.0	82.3 ± 12.7	0.140
Final flexion (°)	126.3 ± 8.0	128.9 ± 7.2	123.5 ± 10.0	0.050
Final extension (°)	−1.3 ± 2.6	−1.7 ± 3.4	−2.0 ± 2.9	0.349
Δ%MA (%)	5.13 ± 13.21	1.53 ± 13.26	4.26 ± 9.62	0.786
ΔJLCA (°)	0.57 ± 1.83	−0.40 ± 3.03	0.40 ± 1.57	0.603

Revision was defined as conversion to UKA or TKA

For %MA, positive Δ values indicate loss of correction from plate removal to final follow-up. ΔJLCA was calculated as the final follow-up value minus the plate removal value

Continuous variables were compared using the Kruskal–Wallis test; categorical variables were compared using the chi-squared test

*JOA* Japanese Orthopaedic Association, *%MA* percentage mechanical axis, *JLCA* joint line convergence angle, *UKA* unicompartmental knee arthroplasty, *TKA* total knee arthroplasty

Accordingly, stratification according to postoperative %MA at the time of plate removal revealed no significant differences in revision rate, final clinical outcomes, or postoperative alignment changes within the KL2–3 group.

## Discussion

The most important finding of this study is that factors associated with revision after OWHTO differed according to preoperative disease severity. Specifically, postoperative alignment at the time of plate removal was associated with revision in KL4 knees, whereas exploratory analysis suggested that progression of JLCA may be associated with revision in KL2–3 knees.

Several recent studies have reported favorable long-term outcomes after OWHTO [6, 12, 13]. Factors such as preoperative disease severity, postoperative alignment, and deformity characteristics have been suggested to influence long-term outcomes [3, 6, 14].

However, factors associated with revision in these advanced cases remain insufficiently understood, and direct KL grade-stratified comparisons are limited.

The present study extends these observations by demonstrating that factors associated with revision differed according to KL grade, with postoperative weight-bearing line position being associated with revision in KL4 knees and time-dependent joint line changes potentially being associated with revision in KL2–3 knees.

In the KL4 group, the revision rate was markedly higher than in KL2–3 knees, and multivariate analysis showed that postoperative alignment at the time of plate removal, represented by %MA, was independently associated with revision.

Although TKA is generally considered the standard treatment for KL4 osteoarthritis, OWHTO may still be considered in selected patients who wish to delay arthroplasty [6]. In KL4 knees, the present findings suggest that greater postoperative lateralization of the weight-bearing line was associated with lower revision rates during long-term follow-up. When postoperative %MA at the time of plate removal was stratified into < 60%, 60–65%, and > 65%, the lowest revision rate was observed in the > 65% group, whereas markedly higher revision rates were seen in knees with < 60% alignment. These findings suggest an association between lower postoperative %MA, particularly below 60%, and higher revision rates in KL4 knees.

Although postoperative time-dependent increases in JLCA were observed, ΔJLCA was not independently associated with revision in the KL4 group. These findings suggest that long-term outcomes in KL4 knees are more closely associated with the weight-bearing line established in the early postoperative period than with subsequent joint line changes. Furthermore, in the present study, lateralization exceeding 65% was not associated with deterioration of final JOA score or with adverse alignment changes, and no clear disadvantages related to excessive lateralization were identified. These findings suggest that moderate postoperative lateralization beyond 65% was not associated with adverse clinical or radiographic outcomes in the present cohort, although the study was not designed to determine an optimal alignment target.

However, excessive lateralization may raise concerns regarding subsequent total knee arthroplasty (TKA) after OWHTO. Previous studies have suggested that conversion TKA after HTO may present technical challenges compared with primary TKA [15]. However, the present study did not evaluate conversion procedures, and the influence of postoperative alignment on subsequent arthroplasty remains unclear.

Importantly, most revision procedures were attributable to recurrence or progression of medial compartment osteoarthritis, supporting the concept that failure after OWHTO is primarily related to recurrent medial loading rather than global osteoarthritic progression. In this context, the markedly lower revision rate observed in KL2–3 knees suggests that when preoperative cartilage degeneration is limited, favorable long-term outcomes may be maintained across a relatively broad range of postoperative alignment. In contrast, in KL4 knees with advanced medial compartment degeneration, lower postoperative correction was associated with a higher likelihood of revision.

In contrast to the KL4 group, exploratory analysis suggested that revision in KL2–3 knees may be more closely associated with time-dependent changes in joint line convergence (ΔJLCA) than with postoperative alignment. These findings suggest that long-term outcomes in KL2–3 knees may be influenced more by subsequent joint line changes than by the initial correction itself.

In the present study, stratification according to postoperative %MA at the time of plate removal did not reveal significant differences in revision rates, clinical outcomes, or alignment changes, suggesting that acceptable long-term outcomes may be achieved across a relatively wide range of postoperative alignment in KL2–3 knees. This relative tolerance may partly explain the low revision rates observed in this subgroup.

Notably, ΔJLCA progression was not accompanied by significant changes in ΔMPTA and showed only a weak association with femoral morphology (mLDFA), suggesting that these changes may not be explained by bony alignment alone. One possible explanation is that ΔJLCA progression may partly reflect underlying biomechanical factors, such as ligamentous laxity; however, this remains speculative because ligamentous laxity was not directly evaluated in the present study. Taken together, these findings suggest that revision in KL2–3 knees may be associated with factors affecting joint line behavior during follow-up rather than with the initial correction itself.

No revision procedures were attributed to symptomatic patellofemoral joint osteoarthritis, even after more than 10 years of follow-up. This finding suggests that PFJ-related changes may have limited influence on long-term revision risk after OWHTO. Similar observations have been reported previously, indicating that radiographic or biomechanical changes of the PFJ do not necessarily translate into clinically relevant failure requiring revision surgery [16].

Therefore, while PFJ-related considerations remain important in surgical planning, the present results suggest that concerns regarding PFJ osteoarthritis alone should not preclude OWHTO when adequate correction and appropriate patient selection are achieved.

Finally, the present results suggest that factors associated with revision after OWHTO differ according to disease severity. Greater postoperative lateralization was associated with lower revision rates in KL4 knees, whereas acceptable long-term outcomes were observed across a relatively wide range of postoperative alignment in KL2–3 knees. These findings highlight the importance of considering disease severity when interpreting factors associated with long-term revision after OWHTO.

## Conclusions

In this long-term study of medial open-wedge high tibial osteotomy, factors associated with revision differed according to Kellgren–Lawrence grade.

In KL4 knees, postoperative weight-bearing line position at the time of plate removal was independently associated with revision, with lower revision rates observed at greater postoperative %MA. However, the present study was not designed to determine an optimal correction target. In contrast, revision was uncommon in KL2–3 knees, and exploratory analysis suggested that time-dependent changes in joint line convergence may be associated with revision; however, this finding requires confirmation in larger cohorts.

These findings improve understanding of factors associated with long-term revision after OWHTO according to disease severity.

### Limitations and future perspectives

Several limitations of this study should be acknowledged.

First, surgical planning at the time of OWHTO was based on a standard target of approximately 70% of the lateral tibial plateau for the weight-bearing line. Consequently, the postoperative alignment at the time of plate removal was not evenly distributed across alignment ranges, and this study was not designed as a prospective comparison of intentionally different alignment targets. Therefore, prospective studies with predefined alignment targets are needed to further clarify the relationship between postoperative alignment and long-term outcomes.

Second, this was a retrospective study conducted at a single institution, and potential institutional biases related to patient selection, surgical technique, implant choice, and postoperative rehabilitation protocols cannot be excluded. In addition, a substantial proportion of patients were lost to follow-up before 10 years. Although baseline disease characteristics, including KL grade and diagnosis, were generally comparable between the included and lost-to-follow-up cohorts, the latter were significantly older and included a higher proportion of male patients. Therefore, despite the comparable baseline disease characteristics, attrition bias cannot be completely excluded and may have influenced the reported survivorship and revision rates.

Third, alignment assessment was based on static standing radiographs. These measurements do not directly reflect dynamic alignment during gait or joint load distribution during daily activities, which may also influence long-term clinical outcomes.

Fourth, intraobserver reliability was assessed in a randomly selected subset of 30 knees and demonstrated good-to-excellent reproducibility for the principal radiographic measurements. However, interobserver reliability was not evaluated, because all measurements were performed by a single observer.

Fifth, revision was defined as conversion to TKA or UKA. Therefore, patients with pain progression, functional deterioration, or dissatisfaction who did not undergo revision surgery were not classified as failures. In addition, some patients may not have undergone revision, because of advanced age, medical comorbidities, low activity level, or personal preference. Consequently, the true incidence of clinical failure may have been underestimated.

Sixth, the variables included in the multivariate analyses were limited, and important factors such as soft tissue degeneration, meniscal pathology, cartilage status, activity level, and postoperative changes in body weight were not fully evaluated. In particular, in the KL4 group, clear preoperative factors associated with ΔJLCA progression could not be identified, suggesting that postoperative joint line changes are likely influenced by multifactorial biomechanical mechanisms. In addition, the number of revision events was limited in the KL2–3 group (*n* = 8), which may have reduced the statistical stability of the multivariate analysis in this subgroup. Therefore, the findings regarding factors associated with revision in KL2–3 knees should be interpreted as exploratory and require confirmation in larger cohorts.

Future prospective multicenter studies incorporating dynamic alignment assessment, advanced imaging, and patient-related factors are warranted to further investigate the mechanisms associated with long-term failure after OWHTO and to clarify the role of postoperative alignment in different stages of disease severity.

Finally, the influence of plate-removal timing on subsequent alignment changes was not separately analyzed, because the timing was primarily determined by the individual bone-healing process and was additionally influenced by exceptional clinical and patient-related factors.

## Supplementary information

Supplementary Material 1.

Supplementary Material 2.

## Data Availability

The datasets generated and/or analyzed during the current study are not publicly available, because they contain information that could compromise participant privacy but are available from the corresponding author on reasonable request.
