# Supplementary material for: Long-term outcomes and factors associated with revision after medial open-wedge high tibial osteotomy in 274 knees: a Kellgren–Lawrence grade-stratified analysis
Source: Knee Surg Relat Res. 2026 Oct 20;38:50. doi: 10.1186/s43019-026-00351-3 (PMC13681812; doi:10.1186/s43019-026-00351-3)
Supplement: Supplementary file 1 [file 43019_2026_351_MOESM1_ESM.docx]

**Supplementary Table 1. Multivariate logistic regression analysis for revision in the KL2-3 group**

Dependent variable: Revision (conversion to UKA or TKA)

Independent variables:

postoperative %MA at the time of plate removal,

ΔJLCA, age, BMI

| **Variable** | **Odds ratio** | **95% confidence interval** | **p value** |
| --- | --- | --- | --- |
| %MA at plate removal | 0.99 | 0.95 – 1.03 | 0.62 |
| ΔJLCA (°) | 3.31 | 1.52 – 7.21 | 0.003 |
| Age (years) | 1.01 | 0.95 – 1.08 | 0.71 |
| BMI (kg/m²) | 1.07 | 0.94 – 1.22 | 0.31 |
