# Supplementary material for: Long-term outcomes and factors associated with revision after medial open-wedge high tibial osteotomy in 274 knees: a Kellgren–Lawrence grade-stratified analysis
Source: Knee Surg Relat Res. 2026 Oct 20;38:50. doi: 10.1186/s43019-026-00351-3 (PMC13681812; doi:10.1186/s43019-026-00351-3)
Supplement: Supplementary file 2 [file 43019_2026_351_MOESM2_ESM.docx]

**Supplementary Table 2. Baseline characteristics of included and lost-to-follow-up patients**

| **Variable** | **Included (n=274)** | **Lost to follow-up (n=212)** | **p value** |
| --- | --- | --- | --- |
| Age (years) | 64.69 ± 7.24 | 67.8 ± 7.6 | <0.001 |
| Female / Male | 207 / 67 | 138 / 74 | 0.016 |
| BMI (kg/m²) | 26.53 ± 3.78 | 25.9 ± 3.8 | 0.085 |
| OA / ON | 226 / 48 | 171 / 41 | 0.692 |
| KL4 / KL2–3 | 158 / 116 | 112 / 100 | 0.331 |
